# Sequence Analysis and Modeling of the Repetitive Region of the Long Isoform of Clarinet/CLA-1

**DOI:** 10.17912/micropub.biology.001712

**Published:** 2025-08-19

**Authors:** Benjamin Hunt, Timothy Hunt, Zhao Xuan

**Affiliations:** 1 School of Biology and Ecology, University of Maine, Orono, Maine, United States

## Abstract

The *C. elegans* active zone gene *cla-1* encodes three main isoforms. The long isoform, CLA-1 L, functions beyond regulating synaptic vesicle exocytosis, including synaptic vesicle clustering and endocytic sorting of a transmembrane autophagy protein. CLA-1 L contains a large N-terminal repetitive region. Sequence analysis indicates that this region is enriched with acidic residues and displays disordered structures interspersed with helical sections. While modeling a portion of the repetitive region revealed dynamic transitions between compact and less compact conformations, modeling the entire region suggests a predominantly extended structure. This study sheds light on how CLA-1 L carries out its diverse functions.

**Figure 1. Structural models of the repetitive region of CLA-1L derived from sequence analysis and modeling with AlphaFold 3 and CALVADOS 2:**
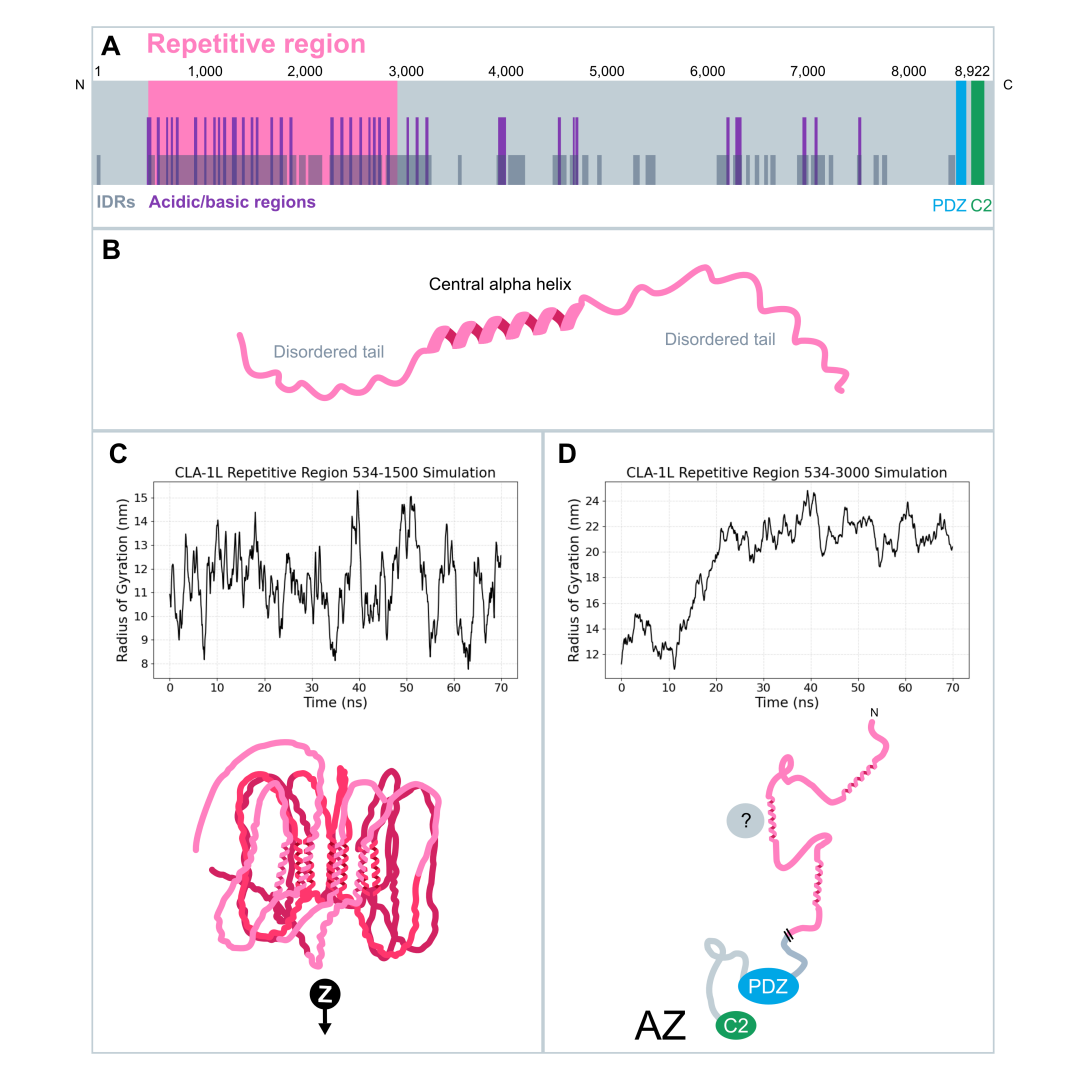
**A.** Sequence analysis of CLA-1 L indicates that intrinsically disordered regions (IDRs) and acidic/basic regions are enriched in the repetitive region. **B.** Each 94-amino acid unit features a central α-helix flanked by intrinsically disordered regions. **C.** Simulation of the first ~10 repeats (residues 534–1500) of the CLA-1 L repetitive region using CALVADOS 2 (above) and AlphaFold 3 (below). AlphaFold 3 modeling suggests that these ten repeats exhibit favorable interactions between individual α-helices in a parallel arrangement, resulting in a more spatially compact conformation. The CALVADOS 2 simulation suggests alternations between compact and less compact conformations. **D.** Simulation of the entire repetitive region using CALVADOS 2 (above) and a hypothetical model of the entire CLA-1 L (below). The upward trend in the radius of gyration suggests there is a loose association between some repeats, resulting in a more expanded conformation of the entire repetitive region. We propose a model where the extensive repetitive region enables CLA-1 L to adopt a more expanded structure. The intrinsically disordered regions within the repeats may allow for versatile interactions with multiple presynaptic components, promoting multifunctional roles of CLA-1 L. This model is supported by spatial localization evidence indicating that the N-terminal region of CLA-1 L (which contains the repeats) resides outside the central active zone (Xuan & Colón-Ramos, 2023).

## Description

CLA-1 has been identified as an essential active zone protein in *C. elegans* , and contributes to synaptic vesicle clustering and the structural organization of synapses (Xuan et al., 2017). The *cla-1* gene encodes three main isoforms that contain common C-terminal PDZ and C2 domains with homology to the vertebrate active zone proteins Piccolo and RIM ( Figure 1A ). The large size (8922 amino acids) and an extensive repetitive region at the unique N-terminus make CLA-1 L the most enigmatic isoform. While the shorter isoforms are required for active zone assembly and proper synapse development, CLA-1 L is essential for synaptic vesicle clustering, supports prolonged neuronal activity, and reduces synaptic depression (Xuan et al., 2017). In addition to a role in facilitating synaptic vesicle exocytosis, CLA-1 L has been shown to bridge synaptic domains to regulate the presynaptic sorting of ATG-9 , the only transmembrane protein in the autophagy pathway (Xuan & Colón-Ramos, 2023). The large size and extensive repetitive region of CLA-1 L hinder traditional methods, such as cloning the genomic region and expressing it as a transgene. Hence, CLA-1 L has been challenging to study, resulting in a lack of research on this protein, particularly in the repetitive region.

To provide insight into the structure and function of the repetitive region, we performed sequence analysis and structural modeling. Our analysis revealed that CLA-1 L harbors a set of 23 tandem 94-amino acid repeats, which represent almost one-third of the total protein ( Figure 1A ). We found that intrinsically disordered regions (IDRs) are enriched in the repetitive region—of the 57 total IDRs identified in the CLA-1 L sequence, 34 (59.6%) are located entirely or partially within this region. IDRs are typically enriched in polar, acidic, or basic residues, which impede the stable folding of their structure (Uversky et al., 2000). Consistent with IDR characteristics, the repetitive region also showed a strong enrichment in acidic/basic regions—25 out of 38 such regions mapped to the repetitive region. In particular, the acidic residue glutamic acid was overrepresented. IDRs lack a fixed three-dimensional structure, enabling them to interact dynamically with multiple protein partners (Dyson, 2016; van der Lee et al., 2014). These compositional features suggest that the repetitive region may adopt a flexible structure, facilitating diverse protein interactions and potentially contributing to the functional versatility of CLA-1 L.

We then used AlphaFold 3 to predict the three-dimensional structures of the first 10 repeats and the full repetitive region of CLA-1 L. We further assessed the compactness of both smaller and larger repeat segments using CALVADOS 2, a coarse-grained model designed to simulate the behavior of intrinsically disordered proteins (Tesei & Lindorff-Larsen, 2022). AlphaFold 3 predicted each 94-amino acid repeat unit to adopt a consistent secondary structure, consisting of a central α-helix flanked by two intrinsically disordered tails ( Figure 1B ). When modeling the first 10 repeats of the repetitive region (residues 534 to 1500 in CLA-1 L) using AlphaFold 3, the α-helical cores of adjacent repeats exhibited attractive interactions, leading to a stacked or clustered arrangement in a parallel configuration ( Figure 1C and Movie 1). This model was supported by CALVADOS 2 simulations, which showed that the radius of gyration—an indicator of protein compactness—fluctuated, suggesting dynamic transitions between more compact and less compact conformations (Bagewadi et al., 2023; Lobanov et al., 2008). The oscillatory behavior of the radius of gyration suggests that the repeats do not adopt a well-defined three-dimensional structure, consistent with the region being largely composed of IDRs. The CALVADOS 2 simulation of the entire repetitive region (534-3000aa; ~23 continuous repeats with two gaps and two truncated repeats) suggests an extended structure ( Figure 1D ), as indicated by the upward trend in the radius of gyration. AlphaFold 3 modeling of the entire repetitive region predicted low-confidence structures of α-helical cores distributed in clusters. Since AlphaFold 3 remains fundamentally limited to stable, well-structured proteins that adopt a relatively fixed form ( *AlphaFold 3* , n.d.; Riley et al., 2023), we favor the model that the repetitive region adopts a more extended conformation as the repeat number increases ( Figure 1D ). Furthermore, an extended conformation is more plausible, as we previously observed that the C- and N-termini of CLA-1 L localize to different spatial regions of the presynapse, with the C terminus being concentrated in the active zone and the N-terminus, containing the repetitive region, reaching into the lateral active zone or periactive zone (Xuan & Colón-Ramos, 2023).

Repetitive regions occur in 14% of all proteins and high-incidence repeats are associated with unique eukaryotic functions (Marcotte et al., 1999). One example of a protein with a functionally significant repetitive region is ankyrin-1 (ANK1). It has 24 tandem 33-amino acid ankyrin repeats, which play an important role in linking spectrin-actin base membrane skeletons to the plasma membrane (Gallagher et al., 1997; Lux et al., 1990). Since CLA-1 L shares its C-terminus with shorter isoforms, and deletion of the long isoform alone disrupts synaptic vesicle clustering and presynaptic sorting of ATG-9 , this suggests that the unique N-terminus of CLA-1 L—composed largely of a repetitive region—plays a key role in these processes. Our sequence analysis revealed that the repetitive region of CLA-1 L is enriched in IDRs. Thus, IDRs may provide structural features that enable CLA-1 L to carry out its diverse functions in synaptic vesicle clustering and the endocytic sorting of ATG-9 . First, IDRs facilitate liquid–liquid phase separation, an emerging mechanism underlying various forms of presynaptic compartmentalization, including synaptic vesicle clustering (Choi et al., 2024). A recent study showed that the giant active zone scaffold protein Piccolo (~5000 amino acids) undergoes phase separation to extract synaptic vesicles from synapsin condensates and deliver them to the active zone surface upon Ca²⁺ entry—a mechanism that supports synaptic vesicle transport from the reserve pool to the readily releasable pool (RRP) (Qiu et al., 2024). CLA-1 L may maintain synaptic vesicle clustering by regulating phase separation through its IDRs, and its large size may allow it to span distinct synaptic vesicle pools and facilitate synaptic vesicle shuttling between them—critical for sustaining neurotransmission. Second, the structural flexibility of IDRs allows them to modulate their function in response to specific cellular contexts, effectively serving as adaptable hubs that facilitate diverse protein interactions and signaling pathways (Dyson, 2016; van der Lee et al., 2014). In the context of ATG-9 endocytosis, we found that CLA-1 L genetically interacts with periactive zone endocytic proteins such as Eps15/ EHS-1 and intersectin/ ITSN-1 . Double mutants exhibit a synergistic effect on the presynaptic mislocalization of ATG-9 (Xuan et al., 2023). The repetitive region of CLA-1 L, may regulate ATG-9 endocytosis by interacting with endocytic factors at the periactive zone. The α-helices at the center of each repeat unit may serve as semi-rigid cores that provide modular stability. Depending on the cellular context, these α-helices could also mediate interactions with one another or with other presynaptic proteins, thereby coordinating vesicle retrieval from the reserve pool, sorting of endocytic intermediates, and maintenance of the recycling pool. Overall, the large size of CLA-1 L, along with its unique repetitive region, may enable it to serve both a structural role in maintaining synaptic vesicle clustering and a regulatory role in the endocytic sorting of ATG-9 at presynaptic sites. Further studies, such as TurboID-based proximity labeling, will help identify proteins interacting with CLA-1 L, shedding light on the mechanisms by which CLA-1 L performs its multifunctional roles.

## Methods

The protein sequence for the long isoform of Clarinet ( **accessionID W6RTA4-1** ) was retrieved from the UniProt database, which contains existing annotations (e.g., acid/basic regions, disordered regions), derived from UniProt's annotation pipeline that identifies features by utilizing external tools, including: TMHMM, SignalP, Phobius, Coils, and MobiDB-lite ( *UniProt* , 2022). The acid and basic regions were analyzed in Excel to identify the relative composition between different amino acid residues, the percentage of acidic and basic residues in the regions, and the number of aspartic acid and glutamic acid residues in each region. Repetitive region analysis was conducted on the entire sequence using a local installation of T-REKs for the discovery and alignment of novel repeats in sequences using a K-means algorithm (Jorda & Kajava, 2009). The repetitive region annotations for T-REKs were then added to CLA-1 L using Geneious Prime (https://www.geneious.com/). These annotations were leveraged to guide targeted structural predictions for sections of CLA-1 L with AlphaFold 3. Since the accuracy of AlphaFold 3 decreases significantly over 2000 amino acids, the annotations were used to make predictions for a portion of the repetitive region of CLA-1 L. UCSF ChimeraX (Meng et al., 2023) was used to facilitate inspection of individual regions and to create visualization movies. The CALVADOS system was utilized to simulate the repetitive region of CLA-1 L using the methods provided by Tesei and Lindorff-Larsen with three replicates for each section (Tesei & Lindorff-Larsen, 2022). Scripts available at: https://github.com/XuanLab123/ CLA-1 L

## Data Availability

Description: A movie created using UCSF ChimeraX to visualize the structural model of the first ten repeats (aa 534-1501) of CLA-1L.. Resource Type: Audiovisual. DOI: https://doi.org/10.22002/78d7k-v8417
